# Assessing the effectiveness of online emotion recognition training in healthy volunteers

**DOI:** 10.1098/rsos.230372

**Published:** 2023-09-27

**Authors:** Zoe E. Reed, Steph Suddell, Andy Eastwood, Lilian Thomas, Imogen Dwyer, Ian S. Penton-Voak, Christopher Jarrold, Marcus R. Munafò, Angela S. Attwood

**Affiliations:** ^1^ School of Psychological Science, University of Bristol, Bristol BS8 1QU, UK; ^2^ MRC Integrative Epidemiology Unit, University of Bristol, Bristol BS8 1QU, UK; ^3^ National Institute for Health Research Bristol Biomedical Research Centre, University Hospitals Bristol NHS Foundation Trust and University of Bristol, Bristol BS8 2BN, UK; ^4^ Trinity College Institute of Neuroscience, Trinity College Dublin, College Green, Dublin 2, Ireland; ^5^ Psychology, Department of Social Sciences, UWE, Bristol BS16 1QY, UK

**Keywords:** emotion recognition, training, generalizability

## Abstract

Facial emotion recognition (ER) difficulties are associated with mental health and neurodevelopmental conditions, including autism and poorer social functioning. ER interventions may therefore have clinical potential. We investigated the efficacy of ER training (ERT). We conducted three online studies with healthy volunteers completing one ERT session. Studies 1 and 2 included active and control/sham training groups and tested the efficacy of (i) four-emotion ERT (angry, happy, sad and scared) (*n* = 101), and (ii) six-emotion ERT (adding disgusted and surprised) (*n* = 109). Study 3 tested generalizability of ERT to non-trained stimuli with groups trained and tested on the same stimuli, or different stimuli (*n* = 120). Training effects on total correct hits were estimated using linear mixed effects models. We did not observe clear evidence of improvement in study 1 but note the effect was in the direction of improvement (*b* = 0.02, 95% confidence interval (CI) = −0.02 to 0.07). Study 2 indicated greater total hits following training (*b* = 0.07, 95% CI = 0.03–0.12). Study 3 demonstrated similar improvement across groups (*b* = −0.01, 95% CI = −0.05 to 0.02). Our results indicate improved ER (as measured by our task), which generalizes to different facial stimulus sets. Future studies should further explore generalizability, longer-term effects and ERT in populations with known ER difficulties.

## 1. Introduction

The ability to perceive and recognize facial emotional expressions is an important facet of social cognition [1] and an essential non-verbal tool for interpersonal communication—enabling us to infer the mental states of others [2]. Human social behaviour, such as engaging with or avoiding other people, is also influenced by the perception of emotions [3]. Previous research has suggested that difficulties in this sociocognitive domain (i.e. recognizing others' emotions) is associated with a range of mental health and neurodevelopmental conditions, greater internalizing and externalizing behaviours, and problems in areas such as social competence, academic ability and social skills [4–7]. For example, poorer emotion recognition (ER) has been found in individuals with depression [8,9], and this may be a causal factor in the maintenance of depressive symptoms [10]. Similarly, autistic individuals or those scoring higher on autistic trait measures tend to have lower accuracy for global ER [11–13]. This may have implications for social development and interpersonal skills over time [14]. Developing a training paradigm to improve ER accuracy may have therapeutic benefits for a number of mental health and neurodevelopmental conditions [15]. However, given that autistic individuals, in particular, seem to experience difficulties with global ER (as opposed to specific emotional biases), then developing an intervention for ER, targeting autistic individuals specifically, is important. While some studies have suggested emotion-specific difficulties (e.g. with emotional expressions other than happy), meta-analyses in this area do not report strong evidence for emotion-specific difficulties, but instead highlight global difficulties as the area to target for autistic individuals experiencing these difficulties [14,16]. Previous research has highlighted the potential for such interventions in autism, but many lack a strong evidence base and it is unclear whether training gains generalize beyond trained stimuli [17].

Previous research has shown that is possible to attenuate atypical ER *biases* (i.e. a tendency to consistently interpret ambiguous facial expressions as a particular emotion), which are present in several mental health conditions, using a brief digital training task. For example, biases have been found towards perceiving sadness in anxiety and depression, and anger or disgust in alcohol use disorder [15,18]. Training studies in anxious and depressed samples have shown decreased biases to negative emotions post-training, and that this decrease is associated with some improvement in mood and quality of life, although these therapeutic benefits are inconsistent across studies [19,20].

Using a similar approach, we have developed a computer-based task that measures the ability to *recognize* emotional expressions in faces (i.e. identifying the correct emotion for a facial expression) [21]. Such a task could serve as an intervention for populations who are known to have difficulties in global ER (as opposed to *biased* recognition seen in depression), such as in autistic individuals. However, it remains unclear whether this ER task can be adapted to *improve* recognition of emotional expressions as well as measure them, similar to the tasks previously developed that can modify biases in emotional expressions. Here, we assess whether our adapted training task can improve ER. This initial evidence would be an important step towards the development of a new ER intervention, that may be beneficial for autistic individuals, and others, that have difficulties in this area. There are other digital ER interventions that already exist described in detail elsewhere [22–26]. However, the primary difference between our task and these existing interventions is the unique way that the stimuli have been developed (i.e. by combining multiple people's facial expressions to form a set of prototypical stimuli with many levels of intensity). Development of these stimuli was part of a large body of work creating a set of stimuli to use across various tasks, one of which is the set used in the aforementioned bias tasks which have a strong evidence base. In addition, our task also includes up to six emotions whereas some other tasks only include four. Many other studies in this area also do not examine generalizability and not all use real faces. Finally, many previous studies have been conducted in very small samples. Our task addresses these limitations in this study and will additionally be part of a wider tailorable intervention covering different aspects of ER which has been informed by discussions with stakeholders.

When developing a training paradigm, as well as improving the outcome of interest, it is essential that effects generalize to wider contexts outside of the specific study conditions. The majority of ER research uses static facial stimuli [27] with some research demonstrating that ER training (ERT) can generalize outside of the initial training setting and to different stimuli [28,29]. Therefore, it would be useful to also assess if that is the case for our ERT.

Here we investigate whether an ERT task we have developed improves ER accuracy. We conducted three online experimental studies. The protocols for each study were pre-registered on the Open Science Framework. Study 1 (https://osf.io/x4kh3) aimed to test the effect of a four emotions (angry, happy, sad and scared) version of our ERT task. Study 2 (https://osf.io/drby2) tested the same task with six emotions (additional emotions of disgusted and surprised). Study 3 (https://osf.io/bpzcj) tested whether training effects generalized to novel (non-trained) faces. We hypothesized that there would be an increase in ER accuracy (measured by total hits) after active ERT compared to sham training (studies 1 and 2). We also hypothesized that training effects would be present and comparable between two groups trained on previously seen (congruent) versus two groups trained on previously unseen (incongruent) stimuli (i.e. that effects would generalize to non-trained faces). Secondary analyses in these studies included examining hits, false alarm rates and sensitivity scores across the individual emotions, as well as examining various subjective outcomes to further our understanding of our training effects.

## 2. Study 1

### 2.1. Methods

For all studies, healthy volunteers were recruited through the online recruitment platform Prolific (https://www.prolific.co/) and data were collected via Gorilla, an online experiment builder (http://www.gorilla.sc/) [30]. All studies consisted of a single session of ERT (the whole session took place in one session for each study, lasting approximately 15 min). Participants completing one study were prevented from participating in the other studies. Participants were paid £4 for study 1, £2.50 for study 2 and £2.20 for study 3, based on estimated time to complete the study and resources available. All participants were paid at approximately the rate suggested by Prolific at the time. Ethics approval was obtained from the School of Psychological Science Research Ethics Committee at the University of Bristol for all studies.

#### 2.1.1. Participants

We recruited 110 healthy volunteers randomized in a 1 : 1 ratio to one of two training groups (active or sham). To participate in this study, participants had to be aged 18 or over and fluent in English. Exclusion criteria included ever being diagnosed with any mental health condition, currently using psychiatric medication, and having an uncorrected visual impairment (including colour blindness). Screening was based on self-report within the participants' Prolific profiles and screening questions were also asked within the study to verify eligibility.

Sample size was determined based on a previous study reporting an effect size of *d* = 1.08 for the effect of emotional *bias* training on the perception of happy faces [20]. We used a more conservative effect size of *d* = 0.70 to take account of the possibility that the original observed effect size may have been inflated as initial studies tend to report inflated effects if evidence of an effect is based on crossing a threshold of statistical significance (e.g. *p* < 0.05) [31]. We calculated that, at an *α* level of 5% for a two-tailed independent means *t*-test, 110 participants would provide 95% power to detect an effect size of *d* = 0.70, 90% power to detect an effect size of *d* = 0.63 and 80% power to detect an effect size of *d* = 0.54.

#### 2.1.2. Study procedure

**Figure 1.**
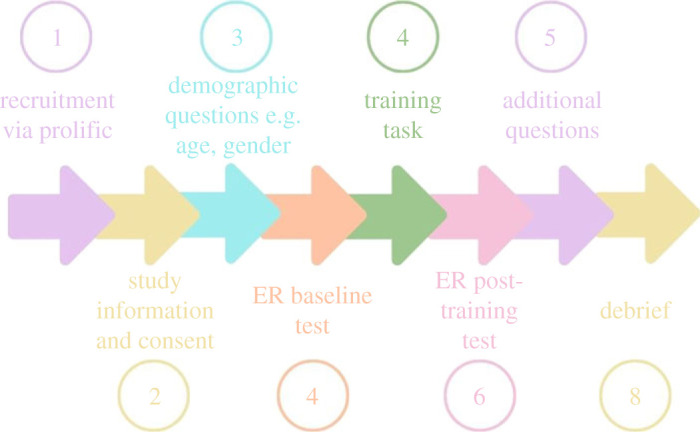
Study session overview. All steps occurred in a single session.

The study procedure is summarized in figure 1 and is similar across all studies. Demographic information on age and gender were collected. All participants completed an initial baseline ER four alternative forced choice (4AFC) test, measuring recognition of angry, happy, sad and scared expressions in male facial stimuli. Participants in the active group then completed a similar ER 4AFC training task in which they received feedback as to whether they had responded correctly or not. If they responded incorrectly, they had to keep responding until they selected the correct emotion. Participants in the sham training group completed a 4AFC training task with feedback, but the stimuli consisted of boxes displaying different colours rather than facial stimuli and participants were asked to select which colour they thought was being displayed. After completing their respective training tasks, participants completed a final ER 4AFC test and questions on subjective outcomes and the positive and negative affect schedule (PANAS). All stimuli in the test and training tasks were presented in a random order and were the same for each individual and across the tasks (i.e. all faces were the same in testing and training tasks).

#### 2.1.3. Emotion recognition test: baseline and post-training

**Figure 2.**
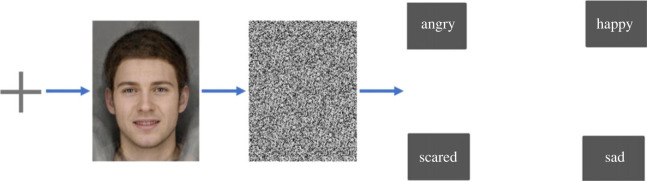
Example of emotion recognition task with happy emotion facial stimuli: first a fixed cross is shown for 800 ms, then an image of a face displaying an emotion for 150 ms, which was backwards masked for 250 ms and finally a screen with the four emotions (angry, happy, sad and scared) is shown. The participant selects an emotion as their response. Facial stimuli are computer generated by averaging photos of 12–15 individuals and therefore do not show a real person.

The task used as the baseline and post-training ER tests presented four facial emotional expressions (angry, happy, sad and scared). There were 15 levels of intensity presented for each emotion, resulting in a total of 60 trials. Images of facial expressions were shown for 150 ms with backward masking. On the next screen participants were asked to select the descriptor that best described the emotion displayed and there was no time limit for this. Presentation of the facial stimuli was randomized. Images used were of the same (male) individual. Figure 2 displays a trial schematic of the ER test.

#### 2.1.4. Active emotion recognition training task

The active training group completed a training version of the ER 4AFC test described above. The procedure was the same as for the test, except that the face was shown for 1 s and response feedback was provided. The participant had to keep responding until they answered correctly.

#### 2.1.5. Sham (colour) training task

The sham training group completed a similar training task to the active group, again with response feedback. However, the stimulus set consisted of coloured blocks instead of facial stimuli. The colours used for these blocks were blue, red, green and yellow for the 4AFC task. These colours were presented at the same number of levels of intensity as facial stimuli, on a greyscale (i.e. ranging from grey through to the respective colour in 15 increments), and participants were asked to select which colour they thought was presented.

#### 2.1.6. Subjective outcomes

Participants were asked the following additional questions related to the study as a whole: ‘Did you find the task fatiguing?’, ‘Did you find the task interesting’ and ‘did you find the task challenging?’ Participants were asked to provide responses on a scale of zero to 100, where zero indicated ‘not at all’ and 100 indicated ‘very much so’.

In addition, participants were asked to provide responses to 20 items from the PANAS [32]. These items were ‘interested’, ‘distressed’, ‘excited’, ‘upset’, ‘strong’, ‘guilty’, ‘scared’, ‘hostile’, ‘enthusiastic’ ‘proud’, ‘irritable’. ‘alert’, ‘ashamed’, ‘inspired’, ‘nervous’, ‘determined’, ‘attentive’, ‘jittery’, ‘active’ and ‘afraid’. Participants indicated the extent to which they currently felt each of the emotions/feelings from the options ‘very slightly or not at all’, ‘a little’, ‘moderately’, ‘quite a bit’, ‘extremely’.

#### 2.1.7. Outcome measures

For statistical analyses, our outcomes included: (i) total hits in the baseline and post-training tasks, i.e. the total number of correct responses (indicative of ER accuracy), (ii) hits per emotion, (iii) false alarms per emotion (i.e. the number of times a particular emotion was selected when this was not the correct response), and (iv) sensitivity scores per emotion using the signal detection theory Aprime (A’) index which is a non-parametric estimate of discriminability. Sensitivity scores were calculated using the Dprime function from the R ‘Psycho’ package [33] which calculates a number of indices. We used the A’ measure, which is a non-parametric estimate of discriminability, where values near 1 indicate good discriminability and values near 0.5 indicate poorer discriminability (i.e. closer to chance). The R function uses the Hautus adjustment (adding 0.5 when calculating A’ so that where participants have 1 for hit rate or 0 for false alarm rate the A’ can still be calculated). Hits and false alarm outcomes were converted to proportions for analyses.

After removal of outliers, skewness and kurtosis measures were examined. Histograms of the distribution of total hits as a proportion at baseline and post-training are shown in the electronic supplementary material, figure S1. Skewness and kurtosis measures were within an acceptable range (see the electronic supplementary material, S2 for further detail).

#### 2.1.8. Statistical analysis

All analyses were conducted in R version 4.0.0 or 4.0.2 depending on when analyses were conducted [34]. We compared group differences (active versus sham training) using a linear mixed effects (LME) model with total hits as the outcome and time (i.e. post-training compared to baseline ER test), group and an interaction term for time x group as the predictors, while accounting for between subjects (specified as participant identity (ID)) random variance. To do this, we used the lme4 package in R [35], where fixed effects of time and group were included in the LME model. Finally, random intercepts for each participant were included for the random effects. The LME model is different to the planned ANOVAs included in our pre-registered protocol. We made this change to the analysis plan as this model allows for more control over random (e.g. participant ID) and fixed (i.e. group, time, an interaction term and covariates) factors. It also allowed us to model all data points rather than aggregated data as in an ANOVA.

We conducted secondary analyses using LME models to explore hits, false alarm rates and sensitivity scores across the individual emotions with the same predictors as in the primary outcome model. Finally, we assessed whether each of the subjective ratings of training experience and the PANAS positive and negative scores (from adding together individuals item scores) varied between the two groups by conducting two-tailed independent means *t*-tests. The active and sham tasks were different, so we examined subjective ratings of the tasks to see how participants found them across the groups.

### 2.2. Results

#### 2.2.1. Exclusion of participants

We recruited 110 participants. However, after removing those from analyses that did not meet eligibility criteria (*n* = 5), or whose data were outliers for total hits (below 0.60 and 0.72 for baseline and post-training hits, respectively) (*n* = 4), there were 101 participants included in our analyses (52 in active and 49 in sham groups).

#### 2.2.2. Participant characteristics

Table 1 shows the participant characteristics.

**Table 1.** Study 1 sample descriptives.

	active (*n* = 52)	sham (*n* = 49)
mean age in years (s.d.)	31 (13)	31 (11)
gender (% of males)	65%	53%
mean total hits at baseline (s.d.)	0.81 (0.08)	0.80 (0.07)
mean total hits post-training (s.d.)	0.88 (0.05)	0.84 (0.05)

s.d., standard deviation.

#### 2.2.3. Analysis results

**Figure 3.**
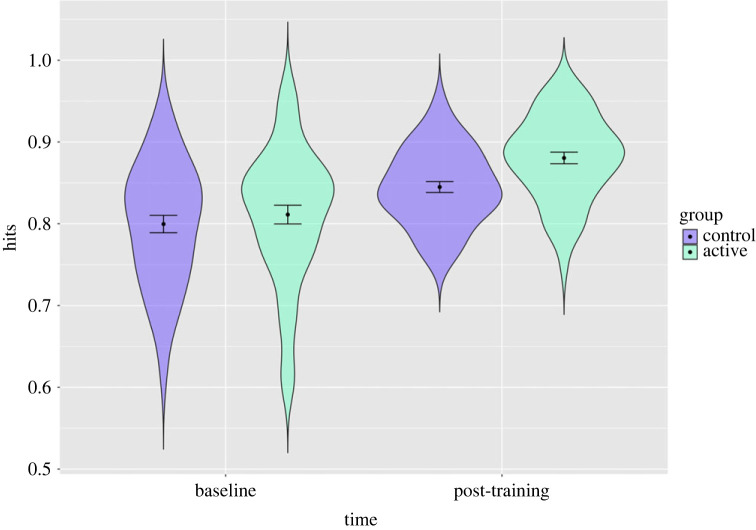
Distribution of participant's total hits with estimates for the active and sham groups at baseline and post-training in study 1. Error bars represent 95% confidence intervals: distribution of participants scores (in terms of proportion of total correct hits) with estimates for each group before and after training and confidence intervals shown. The active group shows a slight improvement post-training.

We found that total hits were greater post-training, with a 6% increase in recognition accuracy in our main effects model (*b* = 0.06, 95% confidence interval (CI) = 0.04–0.08, *p* < 0.001) and greater in the active group (*b* = 0.02, 95% CI = 0.002–0.05, *p* = 0.03) in our main effects model, but there was no clear statistical evidence for the effect of training condition over time in our interaction model (*b* = 0.02, 95% CI = −0.02 to 0.07, *p* = 0.27) (figure 3; electronic supplementary material, table S1).

Results from the LME models for emotion-specific hits, false alarms and sensitivity scores are presented in the electronic supplementary material, tables S2–S4. We found little evidence for an interaction effect between time and group on the number of hits for most emotions in the post-training active sham groups compared to pre-training, except for angry faces where there was an increase (*b* = 0.07, 95% CI = 0.01–0.13, *p* = 0.03). Here, our estimate at baseline for the sham group (i.e. the intercept) is 64% and it is 67% for the active group (percentages reflect the adjusted model estimates). After training this increased to 73% in the sham group and 83% in the active group. For false alarms, there was also little change, apart from a decrease in happy false alarms in the interaction model (*b* = −0.04, 95% CI = −0.07 to −0.01, *p* = 0.02), indicating fewer false alarms. For the sham group at baseline, this was 10% and for the active group this was 9%, decreasing to 9% post-training for the sham group and 4% for the active group. There was little evidence for an interaction effect between time and group on sensitivity scores in the post-training active sham groups compared to pre-training, although we did find weak evidence for an increase for happy (*b* = 0.02, 95% CI = −0.001 to 0.04, *p* = 0.07), where the sham group increased from 0.93 to 0.94 and the active group increased from 0.92 to 0.95, which was probably driven by the decrease in false alarms.

Finally, we did not find any differences between the two groups for most of the subjective ratings or the PANAS negative scores (electronic supplementary material, table S5). There was weak evidence of a difference for the challenging subjective rating, with the active group reporting that this was more challenging than the sham group (active mean = 63 [s.d. = 23], sham mean = 54 [s.d. = 28], *p* = 0.09). The active group also reported slightly lower positive scores than the sham group for the PANAS (active mean = 27 [s.d. = 8], sham mean = 30 [s.d. = 8], *p* = 0.07).

## 3. Study 2

### 3.1. Methods

#### 3.1.1. Participants

We recruited 116 healthy volunteers randomized in a 1 : 1 ratio to one of two groups (active or sham). Exclusion/inclusion criteria were applied as described in study 1, with the additional exclusion criterion of having participated in study 1.

We used the same power calculation to determine the sample size as in study 1 but with an additional increase to participant numbers of 5% based on having to exclude this percentage of participants in study 1 owing to them not meeting the screening criteria within the study.

#### 3.1.2. Study procedure

The procedure was similar to that of study 1 except with a six alternative forced choice (6AFC) design, which additionally included disgusted and surprised emotional expressions in male facial stimuli. The reason for including these additional emotions is that these are the six primary emotions [36] and thus were included in our full suite of stimuli. We initially only included four emotions owing to concerns about the length of the task. However, in study 1 we observed potential ceiling effects and so to make the task more challenging while including a wider array of emotions in study 2, we added in the additional two sets of stimuli for disgusted and surprised and reduced the number of levels of intensity to keep task length similar across the studies. We also collected additional information on education at baseline and questions from the 10-item Autism Spectrum Quotient (AQ-10) [37,38] at the end of the session instead of the PANAS used in study 1.

#### 3.1.3. Emotion recognition test: baseline and post-training

Similar to study 1, in study 2 the ER test was used at baseline and post-training. However, in addition to the four emotions used in Study 1, Study 2 included two additional emotional expressions of disgusted and surprised. Also, to reduce the number of trials owing to the increased number of emotions/colours, participants were only presented with eight levels of intensity for each stimulus (every other level of intensity of the original 15), resulting in a total of 48 trials. The task was the same in all other respects to that used in study 1.

#### 3.1.4. Training tasks

The active training group completed the same task as described in study 1 with the addition of two emotions as described for the test above.

The sham training group completed the same task as described in study 1 as well, with the addition of orange and purple coloured blocks for the 6AFC task with eight increments.

#### 3.1.5. Subjective outcomes

Participants were asked the following additional questions related to the study as a whole: ‘Did you find the task tiring’, ‘Did you find the task interesting’ and ‘did you find the task challenging?’ Participants were asked to provide responses on a scale of zero to 100, where zero indicated ‘not at all’ and 100 indicated ‘very much so’. In addition, participants were asked to provide responses to the AQ-10 (electronic supplementary material, S1).

#### 3.1.6. Outcome measures

For statistical analyses, our outcomes were the same as those for study 1: (i) total hits in the baseline and post-training tasks, (ii) hits per emotion, (iii) false alarms per emotion, and (iv) sensitivity scores per emotion. Histograms of the distribution of total hits as a proportion at baseline and post-training are shown in the electronic supplementary material, figure S2. Skewness and kurtosis measures were within an acceptable range (see the electronic supplementary material, S2 for further detail).

#### 3.1.7. Statistical analysis

Similar to study 1, we compared group differences (active versus sham training) using an LME model with the same predictors. Here, we ran models both excluding and including covariates (for age, gender, education level (as fixed effects)), to improve precision of our effect estimate as these variables are likely to influence our outcome.

We conducted secondary analyses to assess whether the total score on the AQ-10 had any effect on total hits. To do this, we ran a final model including age, gender, education level and the total score on the AQ-10. We ran LME models for each emotion for hits, false alarms and sensitivity scores as exploratory outcomes with the same covariates as in the primary outcome model. Finally, we assessed whether each of the subjective ratings of training experience varied between the two groups by conducting two-tailed independent means *t*-tests.

### 3.2. Results

#### 3.2.1. Exclusion of participants

We recruited 116 participants. However, after removing those from analyses that did not meet eligibility criteria (*n* = 4), or whose data were outliers for total hits (below 0.43 and 0.37 for baseline and post-training hits, respectively; *n* = 2), there were 109 participants included in our analyses (54 in active and 55 in sham groups).

#### 3.2.2. Participant characteristics

Table 2 shows the participant characteristics.

**Table 2.** Study 2 sample descriptives.

	active (*n* = 54)	sham (*n* = 55)
mean age in years (s.d.)	28 (10)	29 (10)
gender (% of males)	67%	67%
education (%)		
degree or equivalent	54%	71%
A-levels or equivalent^a^	28%	22%
GCSEs (grades A* to C) or equivalent^b^	6%	4%
none or unsure	13%	4%
mean total hits at baseline (s.d.)	0.64 (0.08)	0.64 (0.09)
mean total hits post-training (s.d.)	0.75 (0.10)	0.67 (0.10)

^a^A-levels or advanced level qualifications are subject specific qualifications in the UK that are typically completed over 2 years between the ages of 16 and 18 (although can be completed over different time periods and different ages).

^b^GCSEs (General Certificate of Secondary Education) are subject specific qualifications in the UK that are typically completed over 3 years towards the end of secondary school education. A* was the highest possible grade.

s.d., standard deviation.

#### 3.2.3. Analysis results

**Figure 4.**
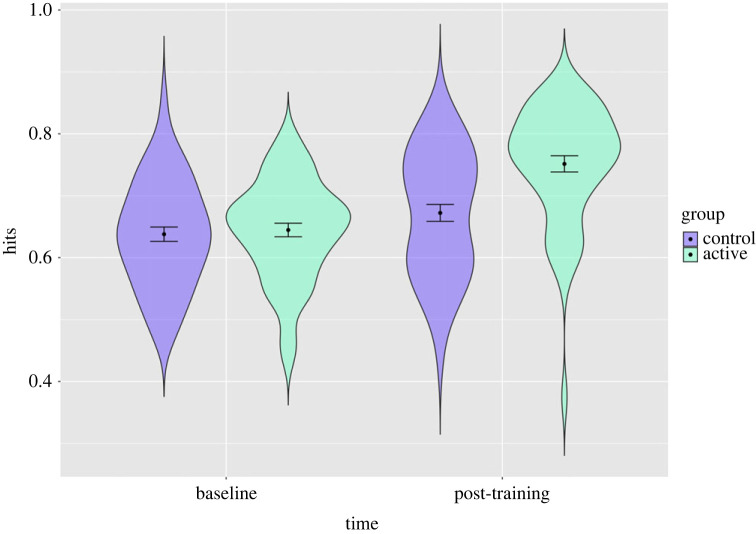
Distribution of participants' total hits with estimates for the active and sham groups at baseline and post-training in study 2. Error bars represent 95% confidence intervals: distribution of participants scores (in terms of proportion of total correct hits) with estimates for each group before and after training and confidence intervals shown. The active group shows greater improvement post-training.

We found that total hits were greater post-training in the active group compared to the sham group in the interaction models excluding and including covariates (including covariates: *b* = 0.07, 95% CI = 0.03–0.12, *p* = 0.002; figure 4; electronic supplementary material, table S6). Here the sham group hits increased from 70% at baseline to 73% post-training and the active group increased from 71% to 81% (percentages reflect the adjusted model estimates). This was unchanged when additionally including AQ-10 scores (*b* = 0.07, 95% CI = 0.03–0.12, *p* = 0.002).

Results from LME models for emotion-specific hits, false alarms and sensitivity scores including covariates are presented in the electronic supplementary material, tables S7–S9. We did not find evidence for an interaction effect between time and group on the number of hits for most emotions in the post-training active sham groups compared to pre-training. However, there was some evidence for increased hits in the active group post-training for scared (*b* = 0.26, 95% CI = 0.17–0.36, *p* < .001), with the sham group increasing from 64% to 67% and the active from 65% to 94% and sad (*b* = 0.07, 95% CI = −0.0002 to 0.13, *p* = 0.05), with the sham group decreasing from 76% to 75% and the active group increasing from 72% to 78%. We also found decreased false alarms for surprised in the interaction model (*b* = −0.05, 95% CI = −0.07 to −0.02, *p* < .001), with the sham group decreasing from 13% to 11% and the active group decreasing from 12% to 5% and sad (*b* = −0.02, 95% CI = −0.04 to 0.002, *p* = 0.07), with the sham group decreasing from 7% to 5% and the active from 8% to 4%. There was no clear evidence for changes in the recognition of other emotions. For sensitivity scores, we found some improvement post-training for the active group compared to the sham group for sad in the interaction model (*b* = 0.04, 95% CI = 0.01–0.05, *p* = 0.01), with the sham group increasing from 91% to 92% and the active increasing from 89% to 94% scared (*b* = 0.13, 95% CI = 0.05–0.20, *p* = 0.001), and (to a lesser extent) surprised (*b* = 0.02, 95% CI = 0.003–0.05, *p* = 0.09), with the sham group increasing from 88% to 89% and the active from 87% to 90%, but no clear differences were observed for other emotions.

Finally, we did not find any meaningful differences between any of the subjective ratings for the two groups (electronic supplementary material, table S10).

## 4. Study 3

### 4.1. Methods

#### 4.1.1. Participants

We recruited 136 healthy volunteers who were randomized to one of four training groups in a 1 : 1 : 1 : 1 ratio. Two of these groups were trained and tested on congruent stimuli (i.e. the set of faces were the same at both training and test, one group for male facial stimuli and the other for female facial stimuli), while the two remaining groups were trained and tested on incongruent (i.e. different) stimuli (one group tested on male and trained on female stimuli and the other tested in female and trained on male stimuli). The same exclusion/inclusion were applied as in study 1, with the additional exclusion criterion of having participated in studies 1 or 2.

Sample size was determined through an *a priori* power calculation based on study 2 results (*d* = 1.1). Based on study 2 being an initial trial of ERT, a more conservative effect size was used (*d* = 0.70). We calculated that, with an *α* level of 5%, 120 participants would provide 95% power to detect an effect size of *d* = 0.70. This was determined for the within-group comparison to assess whether training between the baseline and the post-training test was successful. We recruited an extra eight participants per group (congruent/incongruent) to account for needing to exclude participants and having incomplete datasets. This resulted in a total of 136 participants being recruited.

#### 4.1.2. Study procedure

**Figure 5.**
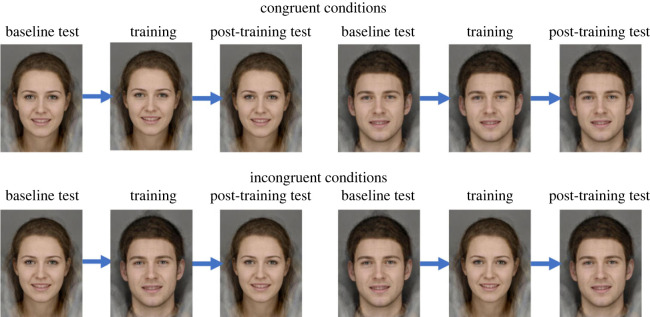
Study 3 training conditions: all four possible conditions are shown. Top-left: female congruent stimuli; top-right: male congruent stimuli; bottom-left and bottom-right: incongruent stimuli (either female–male–female or male–female–male). Facial stimuli are computer generated by averaging photos of 12–15 individuals per set of stimuli and therefore do not show real people.

Study 3 used the same training and test tasks as study 2 (6AFC), however, there was no sham condition and female faces were used in addition to male faces. Instead, all participants received active training on either congruent or incongruent facial stimuli. Participants in the congruent condition were shown the same facial stimuli (e.g. all male or all female faces) in the baseline test, training task and post-training test. Participants in the incongruent condition were shown the same facial stimuli (e.g. male or female faces) in the baseline and post-training tests, but were presented with different stimuli (e.g. male faces if already shown female faces or female faces if already shown male faces) during training. There were four possible training conditions: male congruent, female congruent, male incongruent, female incongruent (figure 5). All four conditions were included to account for any asymmetrical training effects (i.e. if training on male faces transferred to female faces, but not vice versa). However, we were interested in the comparison of congruent versus incongruent and therefore for analyses we collapse these into just two groups representing these two conditions. We also collected responses on Beck's Depression Inventory-II (BDI) scale [39]. This was included for a student project, but data are not reported here as they were not part of our planned analyses. However, we included an attention check item in the BDI, stating: ‘this is to check you are paying attention, please select option 2’. Participants were excluded from analysis if they failed this attention check.

#### 4.1.3. Outcome measures

For statistical analyses, our outcome was total hits in the baseline and post-training tasks. Histograms of the distribution of total hits as a proportion at baseline and post-training are shown in the electronic supplementary material figure S3. Skewness and kurtosis measures were within an acceptable range (see the electronic supplementary material, S2 for further detail).

#### 4.1.4. Statistical analysis

We used an LME model to assess the effects of time (baseline versus post-training) by stimulus congruency (congruent versus incongruent) on proportion of total hits. We ran models without and with covariates for age, participant gender and highest education level. The LME model is different to the planned ANOVAs included in our pre-registered protocol for the same reason as the change for study 1, and to be consistent across studies.

### 4.2. Results

#### 4.2.1. Exclusion of participants

We recruited 136 participants. However, after removing those from analyses whose data were outliers for total hits (below 0.31 and 0.50 for baseline and post-training hits, respectively) (*n* = 5), or who failed an attention check (*n* = 11), there were 120 participants included in our analyses (62 in congruent and 58 in incongruent groups).

#### 4.2.2 Participant characteristics

Table 3 shows the participant characteristics.

**Table 3.** Study 3 sample descriptives.

	congruent (*n* = 62)	incongruent (*n* = 58)
mean age in years (s.d.)	27 (13)	29 (12)
gender (% of males)	53%	53%
education (%)		
degree or equivalent	50%	62%
A-levels or equivalent^a^	18%	28%
GCSEs (grades A* to C) or equivalent^b^	19%	3%
none or unsure	13%	7%
mean total hits at baseline (s.d.)	0.66 (0.10)	0.64 (0.10)
mean total hits post-training (s.d.)	0.77 (0.09)	0.73 (0.08)

^a^A-levels or advanced level qualifications are subject specific qualifications in the UK that are typically completed over 2 years between the ages of 16 and 18 (although can be completed over different time periods and different ages).

^b^GCSEs (General Certificate of Secondary Education) are subject specific qualifications in the UK that are typically completed over 3 years towards the end of secondary school education. A* was the highest possible grade.

s.d., standard deviation.

#### 4.2.3. Analysis results

**Figure 6.**
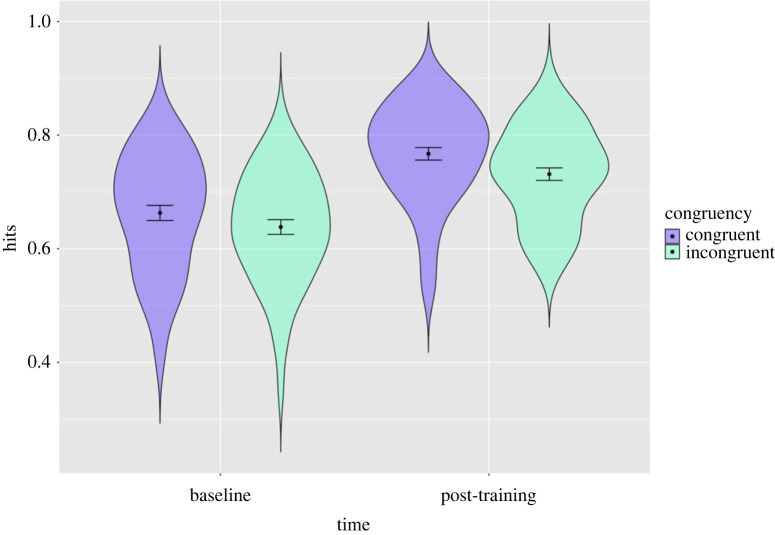
Distribution of participant's total hits with estimates for the congruent and incongruent groups at baseline and post-training in study 3. Error bars represent 95% confidence intervals: distribution of participants scores (in terms of proportion of total correct hits) on the emotion recognition task, per congruency condition. Estimates for each condition before and after training and confidence intervals shown. Participants were either trained on stimuli that were the same (i.e. congruent) or that were different (i.e. incongruent) to the stimuli which they were tested on.

The LME model for proportion of total hits, including covariates, suggested an effect of time in the main effects model (*b* = 0.10, 95% CI = 0.08–0.12, *p* < .001). However, there was no evidence for an effect of stimulus congruency in the main effects model (*b* = −0.02, 95% CI = −0.05 to 0.01, *p* = 0.11), nor evidence of a time by congruency interaction in the interaction model (*b* = −0.01, 95% CI = −0.05 to 0.03, *p* = 0.62), with the congruent group increasing from 72% to 83% and the incongruent group from 70% to 79% (percentages reflect the adjusted model estimates). This suggests that, whilst participants did improve with training, this was not affected by whether participants were tested on the same or different stimuli to which they were trained on (figure 6; electronic supplementary material, table S11).

## 5. Discussion

Taken together, these studies provide evidence that 6AFC ERT improves ER accuracy in healthy volunteers. We demonstrate that it is possible to adapt an ER test into a training task, as has been done previously for emotional bias tests [19,20,40]. In our first study, using only four emotions, we did not find evidence of an improvement post-training in our active group compared to our sham group, although the direction of effect was as expected. However, we observed possible ceiling effects, as participants performed well at baseline, which probably reduced our ability to detect any improvement. In addition, any improvement may have been diluted by practise effects in the sham group. When the difficulty of the task was increased by adding two additional emotions, we found evidence for an improvement post-training in our active group compared to our sham group; thereby supporting the use of this training task as a potential intervention to improve ER. Such an intervention could be targeted at autistic individuals who experience difficulties with ER, for example. In addition, study 3 suggested that this improvement in accuracy may transfer to novel (non-trained) facial stimuli. These findings are consistent with previous research supporting generalizability of facial emotion training [28,29].

In study 2, we additionally observed that improvements were higher for the scared and sad emotions. There was also a decrease in false alarms for surprised and sad, with overall improvements in the sensitivity score for sad, scared and surprised. However, we did not observe these effects across studies so further studies examining emotion-specific hits, false alarms and sensitivity scores are needed to identify if any emotions in particular are impacted more by the training. Finally, we note that including the AQ-10 scores in study 2 did not make a meaningful difference to our results, suggesting that participants' level of autistic traits did not influence our training effect. However, as this was a sample of healthy volunteers, AQ-10 scores are likely to be lower than in autistic individuals where ER difficulties have been previously reported and baseline ER accuracy is likely to be lower [11–13]. Thus, ERT may be more beneficial for autistic individuals, and it would be useful for future research to examine the effectiveness of ERT, in an autistic sample and other populations, that may particularly stand to benefit from improved facial ER accuracy.

In addition, participants in our studies only completed one session of training, indicating a single session of training can provide some improvement in ER. However, it is plausible that multiple sessions of training would result in greater improvement in ER which is sustained over time. Therefore, assessing how this training impacts ER in a multi-session study would be helpful and it would also be useful to identify how many sessions of training would be optimal for delivery of this type of training.

### 5.1. Limitations

There are some limitations to our studies that should be considered. First, while our studies were well powered to detect effects, we did not find evidence for a training effect (i.e. greater improvement in total hits in the active group compared to sham) in study 1—we only observed a trend of improvement. However, as mentioned, this may have been owing to ceiling effects leaving little room for improvement. These ceiling effects probably stemmed from our sample consisting of healthy adults with no known ER difficulties. These improvements were less than those for the active group, but may suggest there is a benefit from simply being exposed to the stimuli (i.e. in the baseline test). However, in study 2, we did observe a training effect, probably owing to the increased difficulty of the task. Therefore, future work with this training should include the six emotions used in studies 2 and 3 and explore this training in populations with ER difficulties. However, it is also worth considering the extent to which the effects we observed in these studies might be owing to mere exposure to the stimuli, and our choice of control task does not allow us to eliminate this possibility. However, in other closely related work on emotional perception training we have employed face-based sham and control tasks, and these show no evidence of exposure-based training effects [19,41]. As the same facial stimuli are used in the training and test phases for the active group, it is plausible that training effects were owing to participants becoming more familiar with the facial stimuli (mere exposure) as opposed to the training component of the task improving underlying emotional processing ability. Mere exposure (i.e. through practise) could deliver a genuine additional training effect that if carried into implementation would not necessarily be problematic. However, if the exposure effect were limited to the face being trained, this could mean inflated estimates of effectiveness. However, as shown here and in previous studies using the same facial stimuli for bias retraining, training effects do transfer to untrained facial stimuli [28,29]. Second, although study 3 demonstrated generalizability to other faces, we have still only tested this for white male and female composite faces and thus generalizability beyond this is unknown. We used this limited stimuli set in these preliminary studies to keep the task length manageable for participants. However, future work will include facial stimuli of different ethnicities and ages and assess the generalizability of training effects across stimulus sets. Third, our study was conducted online which has some limitations, for example, it may be more difficult to be sure that participants actually pay attention to the study and are honest in their responses. However, there are many benefits to running studies online, such as the availability of a large sample pool living in different areas, so a broader sample of participants can be recruited.

### 5.2. Future directions

In addition to the aforementioned future work, this study is also part of the development of a wider intervention which aims to support children, including autistic children, who may have difficulties with ER, and would like to receive support in developing their understanding of others’ emotional expressions. The development of our intervention is not intended to pathologize variation in this area, but to support any individual who wishes to improve their ER. Our wider intervention will incorporate different aspects of ER and open up discussions around why different children may interpret ER within certain contexts differently and encourage mutual understanding, so the onus is not solely on the autistic child. In addition, this study is based on neurotypical ER processes and autistic children may have their own approaches to ER [42], therefore we need to consider this further in future studies. We also aim to create an intervention which is tailorable to move beyond a ‘one size fits all’ approach. Finally, our intervention development has received input from key stakeholders, including autistic adults and children, parents, teaching staff and educational psychologists/therapists and will continue to draw on the expertise of these groups.

## 6. Conclusion

We find ERT improves ER accuracy in a sample of healthy volunteers, and an indication that training gains can transfer to novel faces (as we found no evidence of a difference between the congruent and incongruent conditions). This approach provides a strong basis for future studies around our training task, including testing this in autistic individuals and assessing the effectiveness of a multi-session approach.

## Data Availability

The data and analysis code that form the basis of the results presented here for all studies are available from the University of Bristol's Research Data Repository [43] (http://data.bris.ac.uk/data/), https://doi.org/10.5523/bris.1df0stlnxblc72a13mfnsne3ew. The data are provided in the electronic supplementary material [44].
